# NMR Analysis of the Dynamic Exchange of the NS2B Cofactor between Open and Closed Conformations of the West Nile Virus NS2B-NS3 Protease

**DOI:** 10.1371/journal.pntd.0000561

**Published:** 2009-12-08

**Authors:** Xun-Cheng Su, Kiyoshi Ozawa, Ruhu Qi, Subhash G. Vasudevan, Siew P. Lim, Gottfried Otting

**Affiliations:** 1 Australian National University, Research School of Chemistry, Canberra, Australia; 2 Novartis Institute for Tropical Diseases, Chromos, Singapore; University of Tokyo, Japan

## Abstract

**Background:**

The two-component NS2B-NS3 proteases of West Nile and dengue viruses are essential for viral replication and established targets for drug development. In all crystal structures of the proteases to date, the NS2B cofactor is located far from the substrate binding site (open conformation) in the absence of inhibitor and lining the substrate binding site (closed conformation) in the presence of an inhibitor.

**Methods:**

In this work, nuclear magnetic resonance (NMR) spectroscopy of isotope and spin-labeled samples of the West Nile virus protease was used to investigate the occurrence of equilibria between open and closed conformations in solution.

**Findings:**

In solution, the closed form of the West Nile virus protease is the predominant conformation irrespective of the presence or absence of inhibitors. Nonetheless, dissociation of the C-terminal part of the NS2B cofactor from the NS3 protease (open conformation) occurs in both the presence and the absence of inhibitors. Low-molecular-weight inhibitors can shift the conformational exchange equilibria so that over 90% of the West Nile virus protease molecules assume the closed conformation. The West Nile virus protease differs from the dengue virus protease, where the open conformation is the predominant form in the absence of inhibitors.

**Conclusion:**

Partial dissociation of NS2B from NS3 has implications for the way in which the NS3 protease can be positioned with respect to the host cell membrane when NS2B is membrane associated via N- and C-terminal segments present in the polyprotein. In the case of the West Nile virus protease, discovery of low-molecular-weight inhibitors that act by breaking the association of the NS2B cofactor with the NS3 protease is impeded by the natural affinity of the cofactor to the NS3 protease. The same strategy can be more successful in the case of the dengue virus NS2B-NS3 protease.

## Author Summary

Dengue and West Nile virus infections put an estimated 2.5 billion people at risk. Neither drugs nor vaccines are currently available against these diseases. The non-structural protein NS3 is a protease that, together with the cofactor NS2B, is essential for viral maturation. The NS2B-NS3 proteases of dengue and West Nile viruses are highly homologous and present promising drug targets. Crystal structures of the West Nile virus protease with and without bound inhibitor revealed large structural differences in NS2B, while no crystal structure of the dengue virus protease could be determined with a bound inhibitor. We investigated the structural change in solution and found that the C-terminal segment (CTS) of the NS2B cofactor is prone to dissociation from NS3. In the case of the West Nile virus protease, the CTS of NS2B is mostly associated with NS3, especially in the presence of inhibitors. In the case of the dengue virus protease and in the absence of inhibitors, the CTS of NS2B is mostly dissociated from NS3. Finding drug candidates to inhibit the association of the NS2B cofactor may thus be easier for the dengue virus protease.

## Introduction

West Nile virus (WNV) is a flavivirus related to yellow fever virus, dengue virus, and Japanese encephalitis virus all of which cause human diseases. During infection, the flavivirus RNA genome is translated into a polyprotein comprising of three structural and seven non-structural proteins [1]. The N-terminal part of nonstructural protein 3 (NS3) encodes a serine protease that cleaves the polyprotein into several components. The activity of the NS3 protease (NS3pro) is greatly enhanced by covalent tethering of about 40 residues from the membrane-bound NS2B protein that acts as a co-factor. NS3 is essential for viral replication making it an attractive drug target [2]–[4]. The C-terminal part of NS3 contains a nucleotide triphosphatase, an RNA triphosphatase, and a helicase which have only little influence on the protease activity [5].

**Figure 1:**
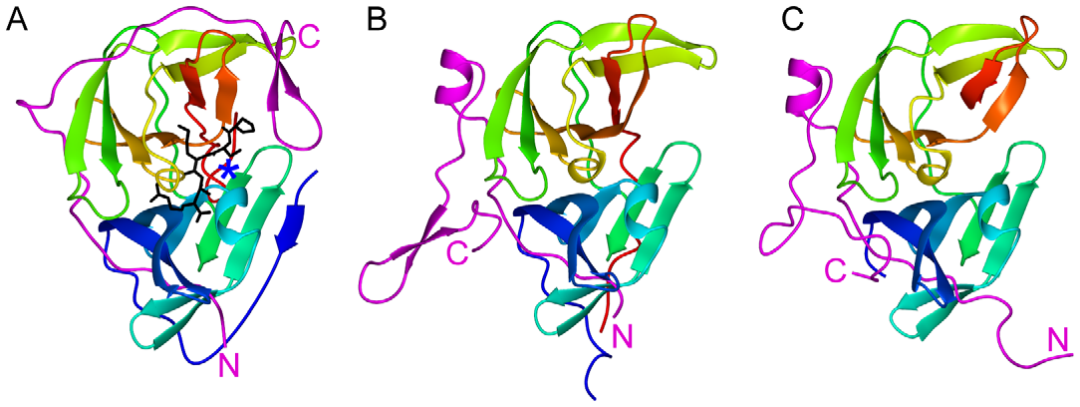
Crystal structures of the West Nile and dengue NS2B-NS3 proteases. NS2B is shown in magenta, with the N- and C-termini labeled. The polypeptide chain of NS3 is shown in rainbow colors ranging from blue (N-terminus) to red (C-terminus). Full-length NS2B comprises 131 residues [1]. All constructs used in crystal structure determinations include the NS2B cofactor segment and exclude the N-terminal 48 and C-terminal 35 residues of NS2B which contain hydrophobic membrane anchors. The constructs used in the present work closely resembled the constructs used for crystallography. In denoting residues 49–60 as the N-terminal segment (NTS) of NS2B and residues 75–96 as the C-terminal segment (CTS) of NS2B, we refer to the cofactor segment of NS2B only. (A) WNV NS2B-NS3pro in the presence of the inhibitor BPTI (PDB accession code 2IJO). The substrate binding site is indicated by the BPTI segment Pro13-Arg17 shown in black. The location of the active-site histidine is labeled with a blue star. (B) WNV NS2B-NS3pro in the absence of inhibitors (PDB code 2GGV). (C) Dengue virus NS2B-NS3pro in the absence of inhibitors (PDB code 2FOM). The present text refers to the protein fold in (A) as closed conformation, while the folds in (B) and (C) are referred to as open conformations.

Crystal structures of WNV NS2B-NS3pro in the absence of inhibitor [6] and in the presence of tetra- and tripeptide inhibitors [7],[8] or bovine pancreatic trypsin inhibitor (BPTI) [6] have been determined. The fold of NS2B is very different in the presence of inhibitors from that in the absence of inhibitor (Figure 1). In all structures, the N-terminal segment of NS2B (residues 52–58) inserts into a β-sheet formed by NS3pro. In the presence of inhibitor, the C-terminal segment (CTS) of NS2B wraps around NS3pro, bringing the C-terminal β-hairpin of the NS2B cofactor in close proximity of the active site. This fold is referred to in the following as the closed conformation. In the absence of inhibitor, the NS2B CTS is located at a very different position far from the active site of the protease. We refer to this fold and any other conformation, where NS2B is disengaged from the substrate binding site, as open conformations. As the NS2B CTS is essential for full catalytic activity of WNV NS2B-NS3pro [2],[9] the closed conformation appears to be a prerequisite for full proteolytic activity.

The fold of the corresponding protease from the closely related dengue virus type 2 (DENV) NS2B-NS3pro construct was also determined by X-ray crystallography in the absence of an inhibitor [7]. NS2B without the CTS results in an inactive protease suggesting that this part of the cofactor forms part of the active site [7]. The fold observed in DENV is remarkably similar to the open conformation of WNV NS2B-NS3pro, suggesting that the open conformation may be the predominant species in solution (Figure 1). Alternatively, the open conformation may be a crystallographic artefact. We undertook the present research in order to address this issue and also because of the failure of high throughput drug screens to identify stable low-molecular weight compounds that bind to the WNV NS2B-NS3 protease specifically and with subnanomolar affinity [10]–[15]. Better understanding of the dynamics of the NS2B cofactor of the protease could be the key towards the design of improved inhibitors and has important implications for the conformational space accessible to the protease when bound to the host cell membrane.

In the following, we present an NMR analysis of the conformational equilibria of the WNV NS2B-NS3 protease in the absence and presence of inhibitors.

## Methods

### Materials

**Figure 2:**
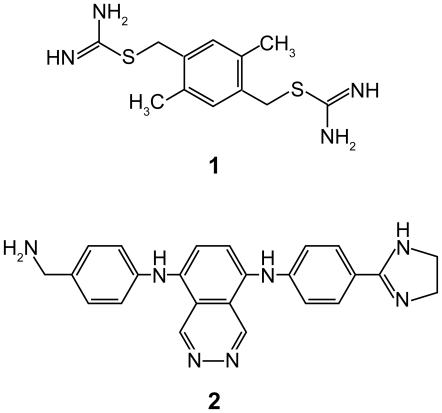
Chemical structures of inhibitors used. Throughout the text, the inhibitors in the top and bottom panels are referred to as compounds **1** and **2**, respectively.

1-oxyl-2,2,5,5-tetramethyl-Δ3-pyrroline-3-methyl) methane thiosulfonate (MTSL) was purchased from Toronto Research Chemicals (North York, Ontario, Canada). Compound **1** (Figure 2) was obtained from Maybridge (Tintagel, UK). Compound **2** was synthesized in-house. The West Nile virus protease construct used contained NS2B covalently linked to NS3pro via a Gly_4_-Ser-Gly_4_ linker [16] as used for crystallization [7]. In addition, Lys96 of NS2B was mutated to alanine to prevent self-cleavage of the protease [17]. In the following, this construct and the unmutated wild type are referred to as NS2B-NS3pro and wt NS2B-NS3pro, respectively. A second construct containing the additional mutation N89C at the C-terminus of NS2B was prepared to provide a thiol group for the attachment of a spin-label. This construct is referred to as NS2B-NS3pro^C^. The N89C mutation was made by site-directed mutagenesis using PCR. Uniformly ^15^N/^13^C- and ^15^N-labeled protein samples of NS2B-NS3pro and five combinatorially ^15^N-labeled samples of the NS2B-NS3 protease without the K96A mutation were prepared as described previously [18]. *In vivo* protein yields were about 9 mg of purified protein per litre of medium. The selectively ^15^N-Ile labeled sample was prepared by cell-free protein synthesis as described previously [18],[19].

### Spin Labeling

The nitroxide radical tag [(1-oxyl-2,2,5,5-tetramethyl-Δ3-pyrroline-3-methyl) methane thiosulfonate, MTSL] was used for spin-labeling. A 0.3 mM solution of NS2B-NS3pro^C^ in 0.5 ml reaction buffer (50 mM Tris, pH 7.6) was treated with 5 equivalents of DTT and washed with DTT-free reaction buffer using a Millipore ultrafilter with a molecular weight cutoff of 5 kD. Reaction buffer was added to a volume of 4 ml. 30 equivalents of MTSL were dissolved in 60 µl acetone and the MTSL solution was added stepwise, mixing the solution well after each addition. The mixture was stirred at room temperature for about 12 hours and subsequently centrifuged to remove undissolved MTSL, followed by concentration to 0.3 ml and washing with 20 mM Tris, pH 7.2.

### NMR Spectroscopy

All NMR spectra were recorded at 25°C on Bruker 600 and 800 MHz NMR spectrometers equipped with cryoprobes. ^15^N-HSQC spectra of the combinatorially ^15^N-labeled samples were recorded in a 20 mM HEPES buffer (pH 7.0) containing 1 mM TCEP. ^15^N-HSQC spectra of a 0.26 mM solution of uniformly ^15^N-labeled NS2B-NS3pro^C^ derivatized with MTSL were recorded in 20 mM Tris, pH 7.2, using the 800 MHz NMR spectrometer with *t*_1max_ = 25 ms and *t*_2max_ = 73 ms. Inhibitor **2** was added to a final concentration of 0.6 mM by adding 3 µl of a 100 mM stock solution in DMSO-d_6_. A 20 mM stock solution of inhibitor **1** in 50% H_2_O/50% DMSO-d_6_ was used for preparing samples containing inhibitor **1**.

^15^N-relaxation rates *R*_2_ were measured at a ^1^H NMR frequency of 600 MHz using the CPMG sequence of Farrow et al. [20] with relaxation delays of 8.8, 17.6, 26.4, 35.2, 44.0, 52.8, 61.6, 70.4, 79.2 and 88.0 ms and a τ_cp_ delay between subsequent 180°(^15^N) pulses of 900 µs. The protein concentration was 0.9 mM in 20 mM HEPES, pH 7.2, 2 mM DTT at 298 K. Experiments with inhibitor were performed with 3 mM inhibitor **2**. The data were analyzed using the program Sparky [21]. The chemical shifts were deposited in the BioMagResBank (accession code 16359).

## Results

### Resonance Assignment of NS2B-NS3pro without Inhibitor

In contrast to NMR spectra in the presence of inhibitors which allowed virtually complete resonance assignments of the ^15^N-HSQC spectra by conventional 3D NMR techniques [18], many of the cross-peaks in the ^15^N-HSQC spectrum of NS2B-NS3pro are broadened beyond detection in the absence of an inhibitor, making the assignment of the NMR resonances challenging (Figure S1) [14],[15],[18]. In order to assign the observable cross-peaks, we used the previously established resonance assignments of the ^15^N-HSQC spectrum of the protease in the presence of inhibitor **1** (Figure 2) and an inhibitor closely related to inhibitor **2** (compound **1** reported by Su et al. [18]) as a starting point. The *K*_d_ values of the inhibitors are in the 10–100 µM range [13],[14]. The inhibitors were in fast exchange between bound and free state, so that titration of the protein with inhibitor yielded a series of ^15^N-HSQC spectra with continuously shifting cross-peaks, allowing tracking of the resonance assignments for the resolved cross-peaks. In addition, combinatorial ^15^N-labeling established the amino acid type associated with each cross-peak.

### Analysis of ^15^N-Ile NS2B-NS3pro

**Figure 3:**
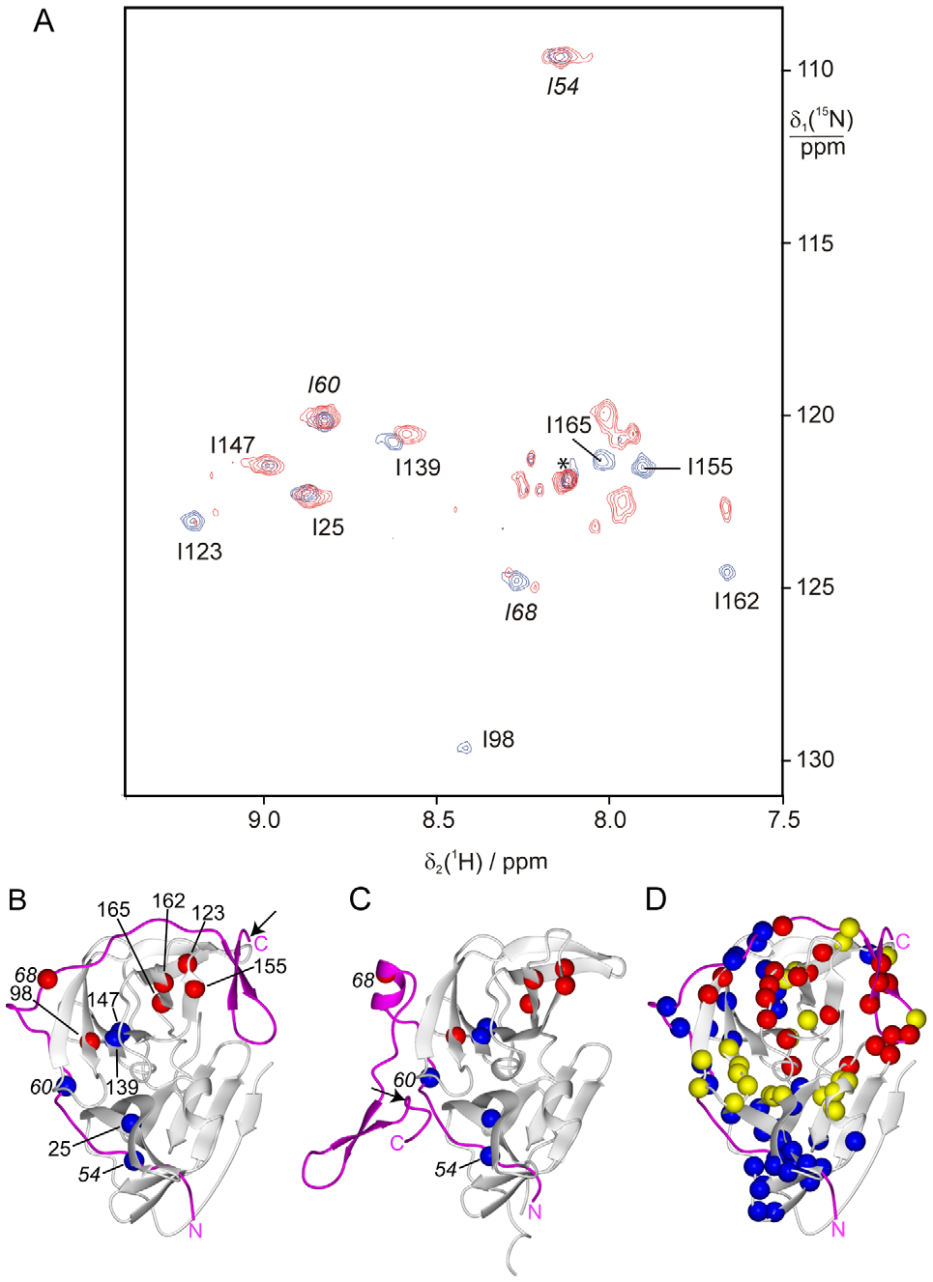
Conformational exchange of NS2B highlighted by inhibitor-induced spectral changes. (A) Superimposition of ^15^N-HSQC spectra of a 50 µM solution of selectively ^15^N-Ile labeled wt NS2B-NS3pro in the presence (blue spectrum) and absence (red spectrum) of 0.3 mM inhibitor **1**. The spectra were recorded at 25°C in a buffer containing 20 mM HEPES, pH 7.0, and 2 mM DTT, using a Bruker 800 MHz NMR spectrometer. The sequence-specific resonance assignments are indicated for the spectrum recorded in the presence of **1**. The star identifies a peak that could not be attributed to any of the isoleucines in the protease. Its relatively narrow line shape suggests its origin from a low-molecular weight impurity. (B) Locations of the isoleucine residues in the crystal structure of the WNV NS2B-NS3 protease (PDB code 2IJO). The nitrogen atoms are drawn as balls, using blue and red colour to identify the residues with, respectively, little and large spectral changes in the ^15^N-HSQC spectra caused by **1**. NS2B is shown in magenta and its N- and C-termini are identified. The isoleucine residues of NS2B are labeled in italics. The arrow identifies the site of Cys89 in NS2B-NS3pro^C^. (C) Same as (B), except for the open conformation (PDB code 2GGV). Only the isoleucine residues of NS2B are labeled for improved visual presentation. (D) Same as (B), except that results obtained with uniformly ^15^N-labeled WNV NS2B-NS3pro are displayed by highlighting selected backbone nitrogens. Red: ^15^N-HSQC cross-peaks were assigned in the presence of inhibitor but seem to be missing in the absence of inhibitor. Yellow: ^15^N-HSQC cross-peaks shifting more than 0.05 ppm in the ^1^H dimension between the spectra recorded with and without inhibitor **1**. Blue: ^15^N-HSQC cross-peaks shifting less than 0.02 ppm in the ^1^H dimension between spectra with and without inhibitor.

As many of the cross-peaks in the ^15^N-HSQC spectrum of uniformly ^15^N-labeled NS2B-NS3pro are overlapped, a selectively ^15^N-Ile labeled sample was prepared for improved spectral resolution. The ^15^N-HSQC spectra of the ^15^N-Ile labeled sample changed greatly in appearance upon addition of the inhibitor **1** (Figure 3A). In particular, all isoleucine residues appeared as single peaks in the presence of **1**, whereas many peaks were missing, significantly shifted or split into several peaks in the absence of inhibitor. Extreme broadening of some but not all lines is a hallmark of chemical exchange of a protein subdomain between different conformations.

Figure 3B shows the locations of the isoleucine residues in the closed conformation, identifying the residues that were strongly or only slightly affected by the presence of inhibitor. The isoleucine residues located in the NS2B CTS or in parts of NS3 in the vicinity of the NS2B CTS are the isoleucine residues most prone to conformational heterogeneity. Too many residues are affected to explain these effects by conformational flexibility of a few active-site residues. The most plausible explanation is that the NS2B CTS assumes multiple conformations in the absence of an inhibitor. We previously reported nuclear Overhauser effects (NOEs) that indicate that the closed conformation of Figure 3A is the prevailing conformation in the presence of inhibitor **1** [18]. In the absence of inhibitor, the closed conformation may still be present but a conformational equilibrium exists that cannot be with the open conformation of Figure 3C as the only additional species, as this would significantly alter the chemical environment of Ile60^NS2B^. The cross-peak of Ile60^NS2B^ is, however, essentially unperturbed.

### Analysis of Uniformly ^15^N-Labeled NS2B-NS3pro

Comparison of the ^15^N-HSQC spectra of uniformly ^15^N-labeled NS2B-NS3pro confirmed the analysis above. Due to spectral overlap, the absence of cross-peaks in the sample without inhibitor could be reliably assessed only for resolved cross-peaks. Nonetheless, we could confirm that the absence of inhibitor led to many missing cross-peaks in the NS2B CTS segment following Glu73 and for segments 73–76, 110–116, 124–130 and 150–153 of NS3 (Figure 3D).

The overall picture is that of extensive dynamics in and around the substrate binding site including, in particular, the C-terminal β-hairpin of NS2B. In contrast, cross-peaks were observable in both states with largely conserved intensities for residues near the N-terminal segment (NTS) of NS2B, as expected for a stable association of the NS2B NTS to NS3. Binding of **1** caused significant chemical shift changes (>0.05 ppm) for a large part of the protein, highlighting the extent of conformational adjustments to the local binding event. Remarkably, the experiment could not identify excessive line broadening for any peaks observable in the segment between residues 53 and 72 of NS2B, whereas the data of Figure 3A–C had identified Ile68 of NS2B as a significantly affected residue. The combined results suggest that, in contrast to the crystal structure data, only the CTS of NS2B following Ser72 is prone to dissociation from NS3pro and that the line broadening of Ile68^NS2B^ is due to a different effect.

The cross-peaks of His51 and S135 of the catalytic triad of NS3 were observable both with and without inhibitor. Therefore, mobility of the side-chain of the active-site histidine, His51, that was reported in a recent crystallographic analysis [8] or other conformational changes in the active site cannot be the cause of the excessive line broadening observed in the absence of inhibitors.

Dissociation of the NS2B CTS from NS3 thus is the main cause for the observed broadening and absence of cross-peaks in the protease without inhibitor. Nonetheless, the NS2B CTS does not exclusively populate a highly mobile random coil conformation (which would result in very narrow peaks) but interacts at least to some extent with NS3.

### ^15^N-Relaxation Measurements

**Figure 4:**
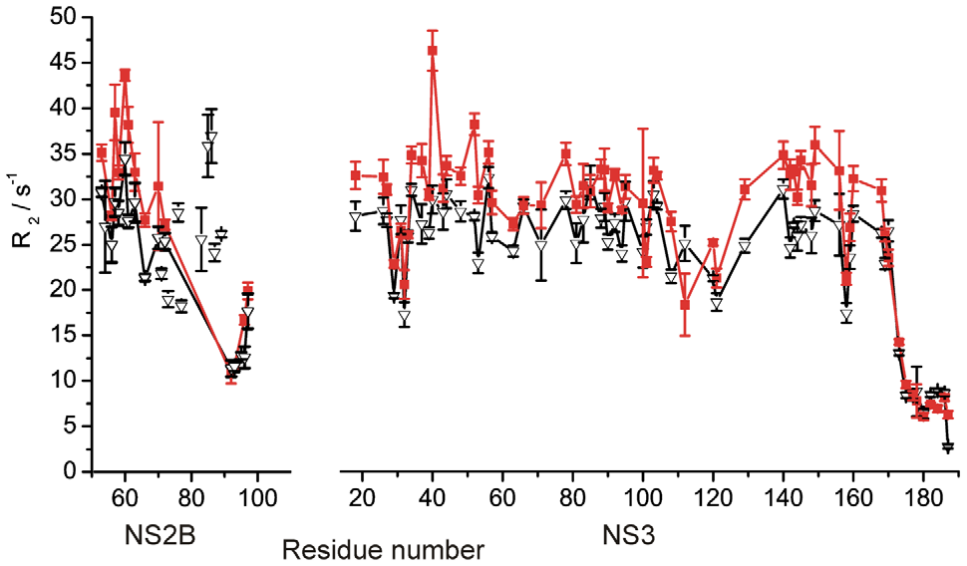
Protein flexibility probed by transverse ^15^N relaxation rates *R*_2_. The data were measured using a 0.9 mM solution of ^15^N/^13^C-labeled NS2B-NS3pro in the presence (black triangles) and absence (red squares) of 3 mM **2**. Data points are plotted versus residue number and connected by lines for improved visual appearance. For NS3, data are shown only for residues for which *R*_2_ data could be measured in both states. All points are shown for NS2B in the presence of the inhibitor **2** as a guide for the mobility of the NS2B CTS. Error bars show the error reported by the fitting routine in Sparky [21].

The flexibility of the polypeptide chain on the subnanosecond time scale was probed by measurement of the *R*_2_(^15^N) relaxation rates of uniformly ^15^N-labeled NS2B-NS3pro. The average relaxation rate was slightly higher in the absence of inhibitor, which may reflect a greater tendency for aggregation (Figure 4). Only the residues C-terminal of Glu173^NS3^ displayed relaxation rates characteristic of a highly mobile random-coil peptide. In contrast, as far as the relaxation rates of NS2B could be assessed, they were similar to those of NS3, both in the absence and presence of inhibitor, suggesting continuous association between the N-terminal segment of NS2B until Ser72^NS2B^ and NS3. In the presence of **1**, the NS2B CTS displayed some of the largest *R*_2_ values, suggesting that the inhibitor did not completely suppress the chemical exchange. Finally, at least ten of the linker residues tethering the C-terminus of NS2B to the N-terminus of NS3 (not shown in Figure 4 due to missing sequence specific assignments) displayed *R*_2_ relaxation rates characteristic of highly mobile segments (below 10 s^−1^), regardless of the presence or absence of inhibitor. This indicates that the peptide linker between NS2B and NS3 presents little hindrance for the dissociation of the NS2B CTS from NS3.

### Paramagnetic Relaxation Enhancements

**Figure 5:**
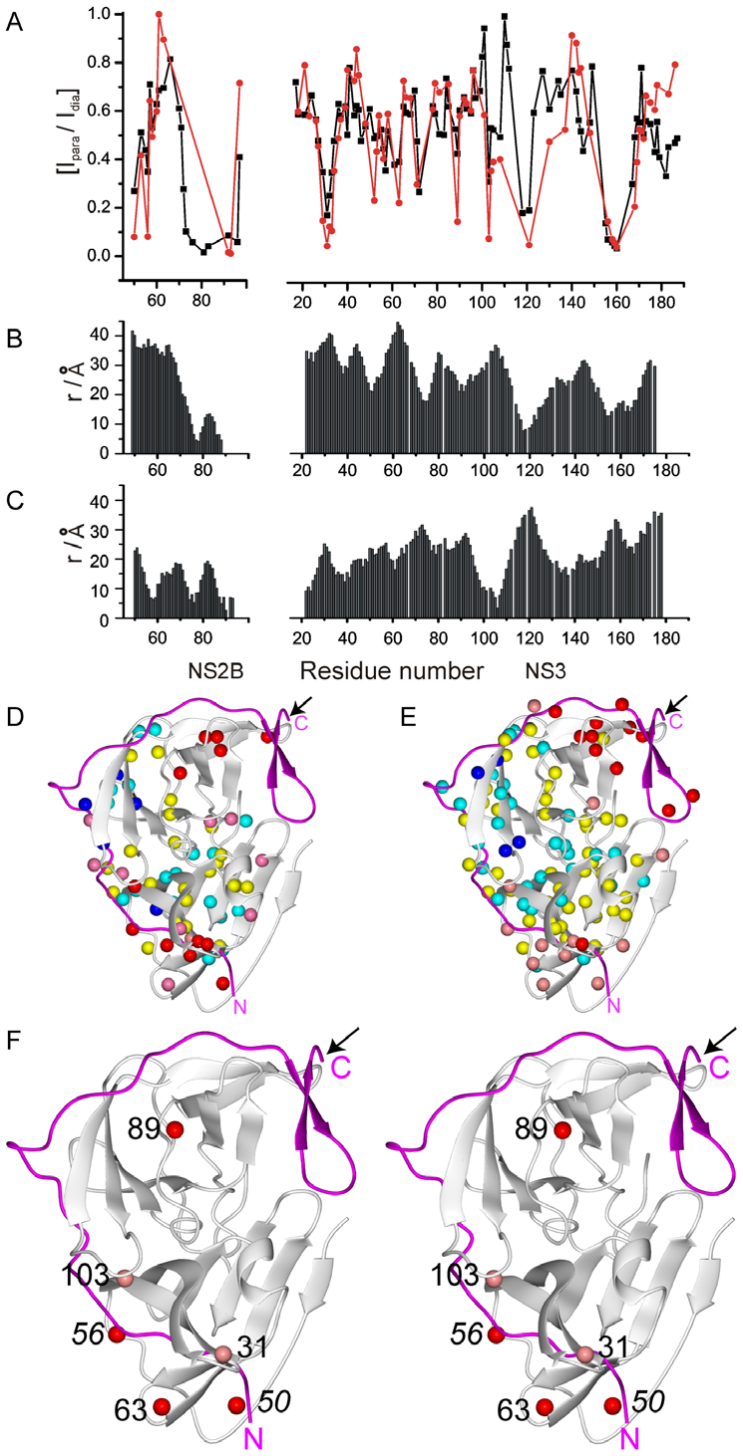
Attenuation of cross-peak intensities by the MTSL spin-label and comparison with proximity to the amide protons in crystal structures of WNV NS2B-NS3pro. (A) Relative peak intensities observed in ^15^N-HSQC spectra of WNV NS2B-NS3pro^C^ with MTSL versus those of WNV NS2B-NS3pro^C^ without spin-label. In order to adjust for uncertainties in protein concentration, the intensity ratios were normalized by setting the largest I_para_/I_dia_ ratio to 1. The black and red data points were measured in the presence and absence of inhibitor **2**, respectively. (B) Distance between the N89C^α^ (as a proxy for the position of the spin-label) and the amide nitrogens in the closed conformation (PDB code 2IJO). (C) Same as (B), except for the open conformation (PDB code 2GGV). (D) Plot of the data in (A), as obtained without inhibitor, on the closed conformation of the WNV NS2B-NS3 protease (PDB code 2IJO). NS2B is shown in magenta. The position of Cys89 in WNV NS2B-NS3pro^C^ carrying the spin-label is identified by an arrow. Amide protons are highlighted with spheres of different colour depending on the I_para_/I_dia_ ratio in (A): 0–0.2 (red), 0.2–0.4 (pink), 0.4–0.6 (yellow), 0.6–0.8 (cyan), 0.8–1.0 (blue). (E) Same as (D), but for the data in the presence of inhibitor **2**. (F) Stereoview of the closed conformation in the representation of (D) and (E). Red spheres identify amide protons of residues for which small concentration dependence in the absence of inhibitor indicates intramolecular PRE effects. Pink spheres identify the location of amide protons for which PRE were significantly concentration dependent, indicating an intermolecular PRE mechanism. NS2B residues are marked in italics.

In order to gain more insight into possible conformational equilibria of the NS2B CTS, we used NS2B-NS3pro^C^ with an MTSL spin-label at Cys89 (location shown in Figures 3B and C and 5D–F). As wild-type WNV NS3pro contains a buried cysteine residue at position 78, we also tested the reactivity of Cys78 in a control experiment by treating wild-type NS2B-NS3pro with MTSL under the same reaction conditions. Analysis by ^15^N-HSQC spectra showed that the cross-peak of Cys78 did not shift or change in intensity, confirming its inaccessibility to MTSL (Figure S1). As expected for a residue without specific long-range contacts, the N89C mutation did not affect the structural integrity of the protease as evidenced by the close similarity of the ^15^N-HSQC spectra of NS2B-NS3pro and NS2B-NS3pro^C^ except for sequential neighbours of residue 89 (data not shown).

Figure 5A shows the results of the spin-labeling experiment. Different ^15^N-HSQC cross-peaks were attenuated differently by the paramagnetic spin-label in a way that is much more readily explained by the closed conformation of Figure 1A (Figure 5B) than the open conformation of Figure 1B (Figure 5C). In particular, the signal attenuations near Cys89^NS2B^ and Ser160^NS3^ were similarly pronounced, indicating that the closed conformation is the predominant species both in the presence and absence of the inhibitor **2**. There were, however, also significant differences between the paramagnetic attenuations observed in the presence and absence of the inhibitor **2** (Figure 5D and E) which must arise either from minor conformational species or from intermolecular effects.

In order to assess possible intermolecular effects, we evaluated the signal attenuations at two different concentrations (Figure S2). A pronounced concentration dependence was observed for the exposed loop region with residues 29–32 and nearby residues (e.g. Gly103), indicating that these regions were significantly affected by intermolecular effects, partly or wholly explaining the apparent discrepancies between the plots of Figure 5D and E for those residues. An increased tendency for intermolecular aggregation in the absence of inhibitor as suggested by the *R*_2_(^15^N) data (Figure 4) also explains why the PRE effects of these residues are greater in the absence of inhibitor (Figure 5A).

The PRE effects of at least four residues (Thr50^NS2B^, Arg56^NS2B^, Gly63^NS3^ and Trp89^NS3^) are pronounced in the absence of inhibitor, depend only little on concentration and cannot be explained by the closed conformation (Figure 5F). We interpret those as intramolecular effects caused by minor conformational species resulting from transient dissociation of the CTS of NS2B from NS3pro. These open conformations must be more heterogeneous than the open conformation of Figure 1B. The relaxation enhancements of all four residues were significantly less pronounced in the presence of inhibitor (Figure 5A), indicating stabilization of the closed conformation by the inhibitor. Therefore, the PRE data support the notion of an equilibrium between a major conformation corresponding to the closed conformation of Figure 1A and an ensemble of transient open conformations that are generally different from the conformation of Figure 1B. Inhibitors shift the equilibrium towards the closed conformation but the equilibrium persists to some extent also in the presence of inhibitors.

## Discussion

The very different folds observed for NS2B in the WNV and DENV NS2B-NS3 protease crystal structures in the presence and absence of inhibitors raise the question of their relative abundance in solution and under *in vivo* conditions, with important implications for the function of these proteases. The present work attributes the extreme broadening of many NMR signals in the absence of inhibitors to the equilibrium between different conformations of NS2B rather than to conformational equilibria restricted to the active-site pocket. As the amide cross-peaks of some of the active-site residues remain unobservable in the presence of inhibitors [17], complete suppression of the conformational exchange in the active-site pocket appears to be more difficult to achieve.

Release of the binding interaction between the CTS of NS2B and NS3pro has implications for the proteolytic activity near the host cell membrane and for drug molecules designed to inhibit proteolytic activity by interfering with the correct association of the NS2B cofactor to NS3.

### Implications for Proteolytic Activity near the Host Cell Membrane

The wild-type WNV polyprotein is associated with the host cell membrane. Also after proteolytic processing of the polyprotein, the NS2B-NS3 protease remains membrane associated via two transmembrane helices N-terminal of the NS2B cofactor part displayed in Figure 1A and B. In addition, the C-terminus of the NS2B cofactor is connected to NS3 by a highly hydrophobic segment of 35 residues [1] (replaced by a Gly_4_-Ser-Gly_4_ linker in our construct) that is also thought to insert into the host cell membrane [22],[23]. This ties the NS2B cofactor to the membrane at either end. Auto-proteolytic cleavage occuring near Lys96^NS2B^ and Lys15^NS3^ excises the segment between NS2B and NS3 *in vitro* [7],[17] but does not affect the N-terminal membrane attachment. As the cleavage site near Lys96^NS2B^ lacks the characteristic recognition sequence of two sequentially neighboring basic residues and is also not conserved in the highly homologous dengue virus NS2B-NS3 protease, cleavage at this site may be inefficient, in which case NS2B would remain tethered to the host cell membrane at either end. Independent of whether the NS2B cofactor is tied to the membrane at one or both ends, there is no reason why the association of NS3pro with the NS2B CTS should be any tighter than that observed in the model system studied here, once the covalent linkage between NS2B and NS3 has been broken.

The association between NS2B and NS3pro appears to be independent of the helicase domain of NS3. In the crystal structure of a DENV NS2B-NS3 construct comprising both the protease and helicase domains of NS3, the interface is centered about residue 68 of the NS3, displacing the N-terminal β-strand of NS3 observed in the structure 2IJO (dark blue in Figure 1A), and the helicase domain makes no contacts with NS2B or the substrate binding site [24]. The same arguments apply to the WNV homologue [22],[23]. It is not clear, however, whether the crystal structure of the DENV NS3 protease-helicase construct is a good model for membrane-associated NS2B-NS3 as it suggests that the helicase domain clashes with the membrane when both ends of NS2B are tied to a planar lipid bilayer.

If the NS3 helicase domain is only loosely associated with the NS3 protease domain, the NS3 protease domain could dissociate from the NS2B CTS to access substrate cleavage sites further away from the membrane surface, although the separation from the NS2B CTS would simultaneously impede its proteolytic activity. The NS2B CTS could, however, follow the NS3 protease domain if its C-terminal membrane attachment is broken by cleavage at the non-canonical site near Lys96^NS2B^, generating a catalytically active NS3 protease that is anchored to the membrane only at the NS2B N-terminus.

### Populations of Different Conformations

The present work shows that, in solution, the closed conformation (or a closely related conformation) is predominantly populated even in the absence of inhibitors. The strongest evidence for this conclusion comes from the similarity of the ^15^N-relaxation with and without inhibitor and from the close similarity between the paramagnetic relaxation enhancements of residues 147–170 with and without inhibitor (Figure 5A). In addition, the PREs indicate that the open conformations detected by the spin-labeling experiment are mostly unrelated to those found in the crystal structures of the WNV and dengue NS2B-NS3 proteases. As PREs strongly depend on the distance from the spin label, they enable the detection of minor conformational species which may be populated by as little as 1%.

The exchange broadening observed for many NMR resonances is also in agreement with the closed conformation as the major species. Chemical exchange of a major conformational species with one or several minor species populated by as little as 5% can lead to the disappearance of cross-peaks if the exchange rate is comparable to the difference in chemical shifts of the conformational states. The appearance of all cross-peaks in the presence of inhibitors can thus be explained, if the inhibitor shifts the conformational equilibrium towards a single conformation (populated by at least 90%). The alternative explanation of a greatly accelerated exchange rate leading to recovery of the cross-peaks is unlikely, as it is difficult to imagine a mechanism by which a low-molecular weight inhibitor would accelerate a major conformational exchange process in the protein.

Considering that inhibitors **1** and **2** do not form van der Waals contacts with NS2B [14],[18], the shift in equilibrium may result from attractive electrostatic interactions with the β-hairpin of the NS2B CTS that carries a sequence of three Asp residues (Asp80^NS2B^-Asp82^NS2B^). Available structures show that inhibitors **1** and **2** [18], BPTI [6] and peptide inhibitors [7],[8] all project positively charged groups towards the β-hairpin of the NS2B CTS. The importance of electrostatic interactions for enzymatic activity of the WNV NS2B-NS3 protease is supported by the observation that increasing concentrations of NaCl decrease enzymatic activity [16].

### Effect of the Linker between NS2B and NS3

All available observations indicate that the structural association of the NS2B CTS with NS3pro is not an artifact of the covalent linker connecting the C-terminus of NS2B with the N-terminus of NS3. (i) *R*_2_ relaxation rates show that at least ten of the linker residues are highly mobile regardless of the presence or absence of inhibitor. (ii) NMR spectra of wt NS2B-NS3 without this covalent link showed no evidence of increased flexibility of the NS2B CTS regardless of the presence of inhibitor (data not shown). (iii) The association of the NS2B CTS with NS3pro is structurally conserved between all three crystal structures of the WNV NS2B-NS3 protease in complexes with different inhibitors, independent of the presence or absence of the linker and for different linker lengths [6]–[8]. All three structures shown in Figure 1 were crystallized with uncleaved linker peptides present, illustrating the freedom of the NS2B CTS to assume the different conformations consistently despite the linker. (iv) Shortening of the linker sequence between NS2B and NS3 by one Gly residue has been shown not to affect the proteolytic activity of the WNV protease [5]. (v) The construct of the DENV NS2B-NS3 homologue used previously for crystallization [7] contained the same number of residues and the Gly_4_-Ser-Gly_4_ segment as the linker peptide in the WNV NS2B-NS3pro construct without preventing enhanced flexibility of the NS2B CTS (see below).

### Comparison with DENV NS2B-NS3 and Implications for Drug Design

Comparison of the present results with previous NMR data of a ^15^N/^13^C-Ile labeled sample of the dengue virus type 2 NS2B-NS3 protease [25] reveals an unexpected important difference. In the case of the DENV protease, in the absence of inhibitor, the ^15^N-HSQC cross-peaks of all five isoleucine residues spanning NS2B from Ile67 to Ile86 appeared at random coil chemical shifts and displayed much narrower line widths than any isoleucine residue in the structured core of NS3pro [25]. The absence of any line broadening in this segment indicates that it is more than 90% of the time dissociated from NS3pro. Nonetheless, the NS2B CTS seems to affect the NMR line widths in the rest of the protein, as much more uniform NMR peak intensities have been reported following cleavage of recombinant NS2B-NS3 protease at Asp81^NS2B^ by endoprotease Asp-N [26].

The association of NS2B to NS3 has important implications for structure-based drug design. First, if the NS2B CTS remains largely dissociated also in the presence of inhibitors, this may explain the persistent difficulties to crystallize the dengue virus enzyme with a bound inhibitor. Second, the relatively close association of the NS2B CTS with NS3 in the case of the WNV NS2B-NS3 protease may make it harder to find inhibitors that suppress the protease activity by preventing the association of the NS2B CTS with NS3pro. In contrast, the closed conformation is an appropriate target for rational drug design and has already been used successfully to identify hits by virtual screening [14],[15].

## Supporting Information

Figure S1 ^15^N-HSQC spectra of 0.3 mM solutions of ^15^N-labeled WNV NS2B-NS3pro(N89C,K96A) with and without MTSL and in the absence and presence of 3 mM **2**. Superimposition of ^15^N-HSQC spectra of WNV NS2B-NS3pro^C^ without (blue spectrum) and with MTSL bound to Cys89 (magenta spectrum) in the (A) absence and (B) presence of the inhibitor **2**. The samples contained 0.3 mM protein in 90% H_2_O/10% D_2_O containing 20 mM Tris buffer (pH 7.2) and 2 mM DTT. The spectra were recorded at 25°C on an 800 MHz NMR spectrometer. The complexes with **2** were prepared by adding 3 µl of a 100 mM stock solution of 2 in d6-DMSO to the protein solution. Resolved cross-peaks are labelled, if they showed significant differences in peak intensities between the samples with and without MTSL. Cross-peaks from NS2B are labelled in italics. A box highlights the cross-peak of Cys78 which is not attenuated by the spin label, demonstrating that this buried cysteine residue did not react with MTSL.
(0.86 MB PDF)

Figure S2 Concentration dependence of the ratio of ^15^N-HSQC peak heights observed for WNV NS2B-NS3pro^C^ with MTSL versus those of unmodified WNV NS2B-NS3pro^C^. Concentration dependence of the ratio of ^15^N-HSQC peak heights observed for WNV NS2B-NS3pro^C^ with MTSL versus those of unmodified WNV NS2B-NS3pro^C^. In order to adjust for differences in protein concentration, scan numbers and receiver gains, the intensity ratios were normalized by setting the largest Ipara/Idia ratio to 1. (A) In the absence of inhibitor. Black squares: 0.26 mM protein. Red circles: 0.13 mM protein. The diamagnetic reference is the 0.26 mM protein in both cases. (B) In the presence of inhibitor **2**. Black squares: 0.26 mM protein with 0.6 mM **2**. Red circles: 0.13 mM protein with 0.26 mM **2**. In both cases, the diamagnetic reference is 0.26 mM protein in the presence of 0.6 mM **2**.
(0.53 MB PDF)
