# Supplementary material for: NMR Analysis of the Dynamic Exchange of the NS2B Cofactor between Open and Closed Conformations of the West Nile Virus NS2B-NS3 Protease
Source: PLoS Negl Trop Dis. 2009 Dec 8;3(12):e561. doi: 10.1371/journal.pntd.0000561 (PMC2780355; doi:10.1371/journal.pntd.0000561)
Supplement: Figure S1 — 15N-HSQC spectra of 0.3 mM solutions of 15N-labeled WNV NS2B-NS3pro(N89C,K96A) with and without MTSL and in the absence and presence of 3 mM 2. Superimposition of 15N-HSQC spectra of WNV NS2B-NS3proC without (blue spectrum) and with MTSL bound to Cys89 (magenta spectrum) in the (A) absence and (B) presence of the inhibitor 2. The samples contained 0.3 mM protein in 90% H2O/10% D2O containing 20 mM Tris buffer (pH 7.2) and 2 mM DTT. The spectra were recorded at 25°C on an 800 MHz NMR spectrometer. The complexes with 2 were prepared by adding 3 µl of a 100 mM stock solution of 2 in d6-DMSO to the protein solution. Resolved cross-peaks are labelled, if they showed significant differences in peak intensities between the samples with and without MTSL. Cross-peaks from NS2B are labelled in italics. A box highlights the cross-peak of Cys78 which is not attenuated by the spin label, demonstrating that this buried cysteine residue did not react with MTSL. (0.86 MB PDF) [file pntd.0000561.s001.pdf]

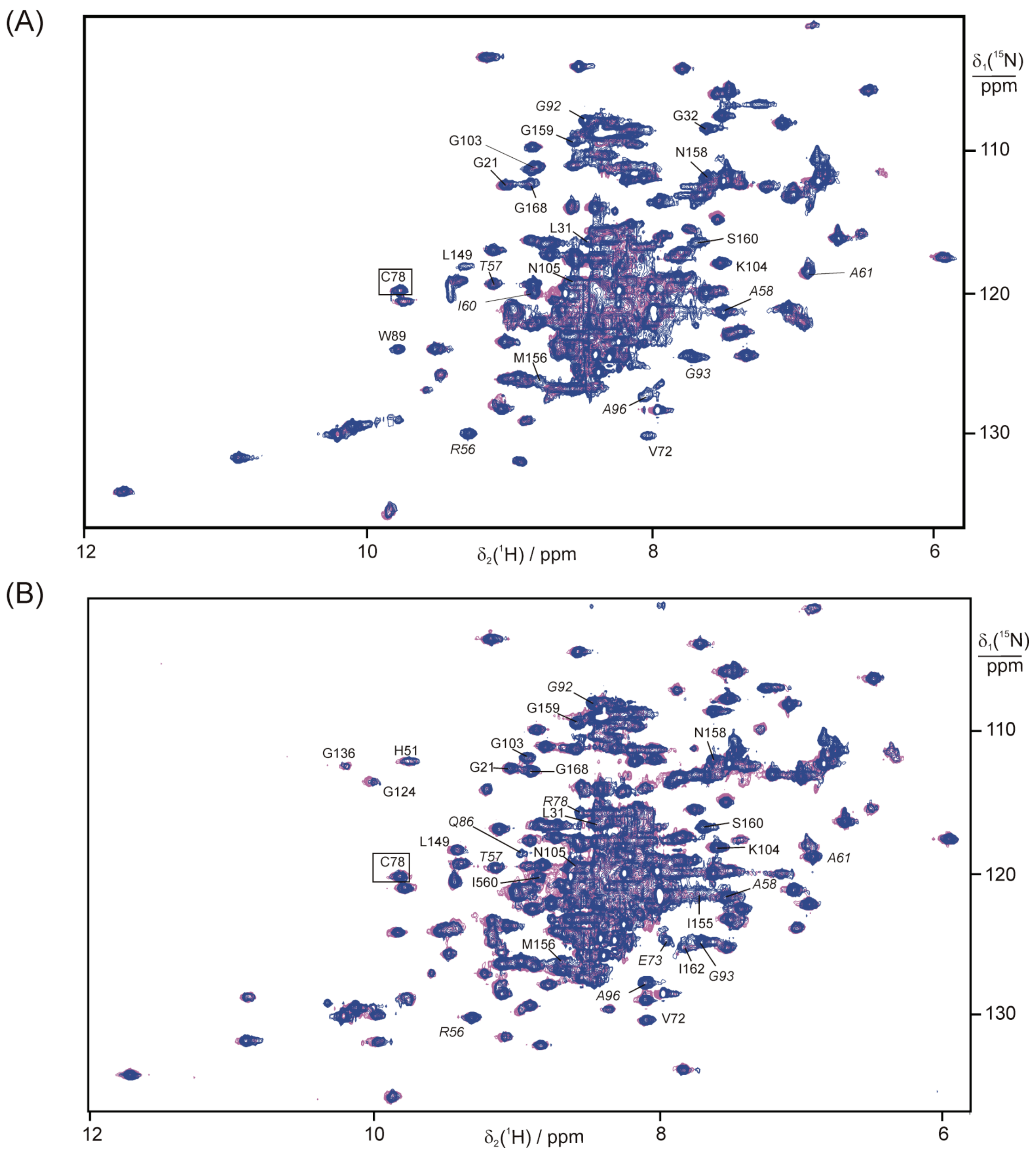

Supporting Figure S1 Superimposition of ^{15}N-HSQC spectra of WNV NS2B-NS3pro^{C} without (blue spectrum) and with MTSL bound to Cys89 (magenta spectrum) in the (A) absence and (B) presence of the inhibitor **2**. The samples contained 0.3 mM protein in 90% H_2O/10% D_2O containing 20 mM Tris buffer (pH 7.2) and 2 mM DTT. The spectra were recorded at 25 ^{o}C on an 800 MHz NMR spectrometer. The complexes with **2** were prepared by adding 3 μl of a 100 mM stock solution of **2** in d_6-DMSO to the protein solution. Resolved cross-peaks are labelled, if they showed significant differences in peak intensities between the samples with and without MTSL. Cross-peaks from NS2B are labelled in italics. A box highlights the cross-peak of Cys78 which is not attenuated by the spin label, demonstrating that this buried cysteine residue did not react with MTSL.
