# Supplementary material for: NMR Analysis of the Dynamic Exchange of the NS2B Cofactor between Open and Closed Conformations of the West Nile Virus NS2B-NS3 Protease
Source: PLoS Negl Trop Dis. 2009 Dec 8;3(12):e561. doi: 10.1371/journal.pntd.0000561 (PMC2780355; doi:10.1371/journal.pntd.0000561)
Supplement: Figure S2 — Concentration dependence of the ratio of 15N-HSQC peak heights observed for WNV NS2B-NS3proC with MTSL versus those of unmodified WNV NS2B-NS3proC. Concentration dependence of the ratio of 15N-HSQC peak heights observed for WNV NS2B-NS3proC with MTSL versus those of unmodified WNV NS2B-NS3proC. In order to adjust for differences in protein concentration, scan numbers and receiver gains, the intensity ratios were normalized by setting the largest Ipara/Idia ratio to 1. (A) In the absence of inhibitor. Black squares: 0.26 mM protein. Red circles: 0.13 mM protein. The diamagnetic reference is the 0.26 mM protein in both cases. (B) In the presence of inhibitor 2. Black squares: 0.26 mM protein with 0.6 mM 2. Red circles: 0.13 mM protein with 0.26 mM 2. In both cases, the diamagnetic reference is 0.26 mM protein in the presence of 0.6 mM 2. (0.53 MB PDF) [file pntd.0000561.s002.pdf]

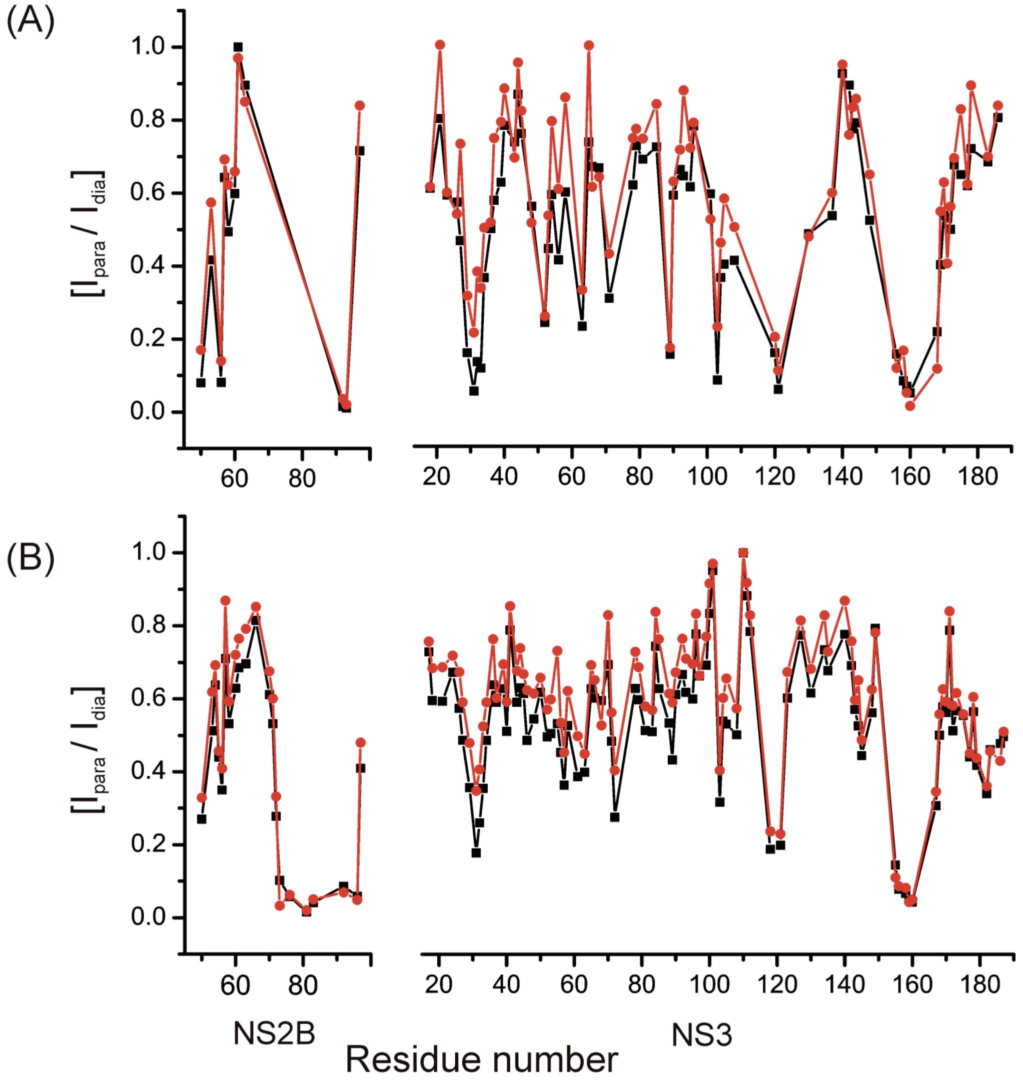

Supporting Figure S2 Concentration dependence of the ratio of ^{15}N-HSQC peak heights observed for WNV NS2B-NS3pro^{C} with MTSL versus those of unmodified WNV NS2B-NS3pro^{C}. In order to adjust for differences in protein concentration, scan numbers and receiver gains, the intensity ratios were normalized by setting the largest I_{para}/I_{dia} ratio to 1. (A) In the absence of inhibitor. Black squares: 0.26 mM protein. Red circles: 0.13 mM protein. The diamagnetic reference is the 0.26 mM protein in both cases. (B) In the presence of inhibitor **2**. Black squares: 0.26 mM protein with 0.6 mM **2**. Red circles: 0.13 mM protein with 0.26 mM **2**. In both cases, the diamagnetic reference is 0.26 mM protein in the presence of 0.6 mM **2**.
